# Use of Guedel Airway as a guide to insert nasogastric tube under general anaesthesia: A simple and logical way

**DOI:** 10.12669/pjms.345.16572

**Published:** 2018

**Authors:** Faraz Shafiq, Faruq Hameed, Khalid Siddiqui

**Affiliations:** 1 Faraz Shafiq, Assistant Professor, Department of Anaesthesiology, The Aga Khan University, Karachi Pakistan; 2 Faruq Hameed, Resident, Department of Anaesthesiology, The Aga Khan University, Karachi Pakistan; 3 Khalid Siddiqui, Assistant Professor, Department of Anaesthesiology, The Aga Khan University, Karachi Pakistan

**Keywords:** Guedel, Nasogastric, Anaesthesia

**Fig.1:**
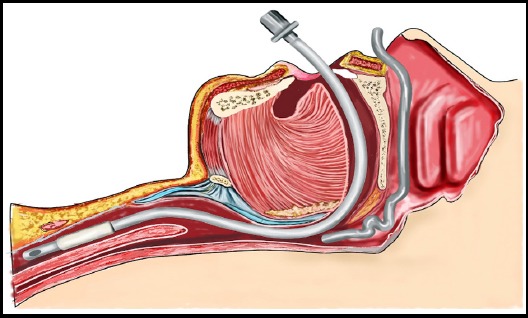
Loss of tone at back of tongue during GA causing resistance to NGT passage

**Fig.2:**
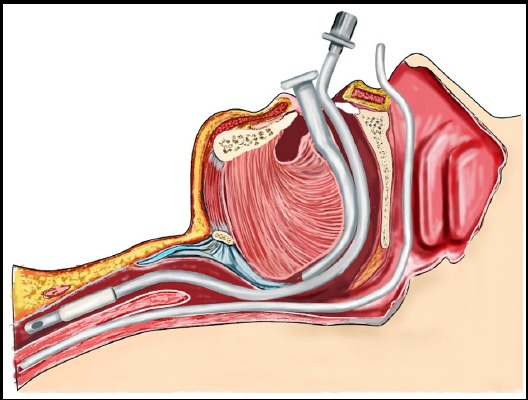
Presence of Guedel airway prevents tongue fall and assisting NGT to pass down

Dear Editor,

We all must have faced difficulty in inserting nasogastric tube (NGT) during general anesthesia (GA). Procedure seems to be very straightforward but has got consequences in terms of failure, which can cause bleeding and alteration in hemodynamic variables. The overall failure rate turned out to be around 50-60%.^1^ The kinking or coiling of distal part of tube in piriform sinus or at level of arytenoids cartilage^2^ is reported to be the frequent cause of this failure. However, it’s still not very clear, as many factors including anatomical variations of oropharynx or type of material used by manufacturer may be responsible. We hypothesized that base of tongue and oropharynx is the possible site of resistance causing difficulty to insert NGT. The situation get worsen in patients having GA due to relaxation of tongue and jaw muscles.^3^ (Fig.1). The use of proper size Guedel oro-pharyngeal airway may be beneficial for NGT insertion in patients under GA. (Fig.2). It does this by preventing tongue fall^4^ minimizing the resistance and likelihood of NGT placement in first pass. We found this technique really useful. However recommend further clinical trial to judge its success alone or as at adjuvant maneuvers for NGT placement.
